# A Blend of Essential Oils Plus Vitamin D3 Combined with *Bacillus subtilis* or with Virginiamycin on Performance, Dietary Energetics, and Carcass Traits of Lambs Finishing Under High Ambient Temperature

**DOI:** 10.3390/ani16162572

**Published:** 2026-08-18

**Authors:** Jesús A. Quezada-Rubio, Alfredo Estrada-Angulo, Beatriz I. Castro-Pérez, Jesús D. Urías-Estrada, Elizama Ponce-Barraza, Lucía de G. Escobedo-Gallegos, Jorge L. Ramos-Méndez, Yesica J. Arteaga-Wences, Alberto Barreras, Alejandro Plascencia

**Affiliations:** 1 Faculty of Veterinary Medicine and Zootechnics, Autonomous University of Sinaloa, Culiacan 80260, Mexico; jesus.quezada.fmvz18@uas.edu.mx (J.A.Q.-R.); alfred_vet@uas.edu.mx (A.E.-A.); laisa_29@hotmail.com (B.I.C.-P.); david.urias@uas.edu.mx (J.D.U.-E.); elizama.ponce@hotmail.com (E.P.-B.); lucia.escobedo@uabc.edu.mx (L.d.G.E.-G.); ramos.jorge.92@outlook.com (J.L.R.-M.); arteaga.yesi.92@hotmail.com (Y.J.A.-W.); 2 Veterinary Science Research Institute, Autonomous University of Baja California, Mexicali 21100, Mexico; beto_barreras@yahoo.com

**Keywords:** essential oils, probiotics, virginiamycin, feedlot lambs, performance, carcass

## Abstract

With the aim of evaluating the effects of combining a natural additive composed of a blend of essential oils plus vitamin D3 (EOD3) with *Bacillus subtilis* (BS) or with the antibiotic virginiamycin (VM) on growth-performance and carcass traits of finishing lambs subjected to high ambient heat load, 48 intact male lambs (initial weight = 33.71 ± 3.21 kg) were used in a feeding trial lasting 61 d. Treatments consisted of supplementing (as a feed basis) a total mixing finishing diet (90:10 concentrate-to-forage ratio) as follows: (1) without feed additives (Control); (2) supplemented with EOD3 (a blend of 100 mg essential oils plus 0.10 mg vitamin D3/kg diet); (3) diet supplemented with EOD3 plus 2 g *Bacillus subtilis* PB6/kg diet to provide 2.2 × 10^8^ CFU (EOD3+BS); and (4) basal diet supplemented with EOD3 plus 25 mg virginiamycin/kg diet (EOD3+VM). The temperature–humidity index (THI) during the experiment averaged 82.52 ± 1.10. Compared to non-supplemented lambs, lambs that received EOD3 alone showed greater (*p* < 0.03) average daily gain (ADG; 15.3%), gain-to-feed ratio (GF; 12.3%), and observed-to-expected dietary NE ratio (5.9%). The combination of EOD3 with BS resulted in identical ADG but a numerically larger DMI (7.1%, *p* = 0.087) than the EOD3 treatment, leading to lower (*p* = 0.024) GF and a lower (*p* < 0.01) observed-to-expected dietary NE ratio. In contrast to our expectation, combining VM and EOD3 did not increase growth response (*p* = 0.69), GF (*p* = 0.49), or dietary energy utilization efficiency (*p* = 0.09) when compared to EOD3 alone. Because the lambs that received supplemental EOD3 alone and their combinations showed greater final weight, hot carcass weight was heavier than in controls; however, no effects were observed on the other carcass variables evaluated, including whole cuts and shoulder tissue composition. Lambs that received EOD3 and their combinations showed a lower relative intestinal mass than controls (*p* < 0.04). This result confirms the role of EOD3 as a tool to minimize the negative impact of high ambient temperature on the performance of fattening lambs. Combining EOD3 with *Bacillus subtilis* or with VM did not improve energy utilization efficiency or carcass traits compared with EOD3 supplementation alone in lambs fattening under high ambient heat load. Further research is needed to better understand the interactions among different additives and to propose more effective supplementation strategies in animals subjected to different environmental conditions.

## Simple Summary

High ambient temperatures adversely affect livestock productivity. Consequently, numerous mitigation strategies have been explored, including the use of supplemental feed additives administered individually or in combination. In the present study, we evaluated whether combining an essential oil blend with vitamin D_3_ (EOD3), previously shown to alleviate the detrimental effects of heat stress on ruminant productivity, with either a *Bacillus subtilis*-based probiotic or the antibiotic virginiamycin could produce synergistic benefits. Forty-eight lambs were exposed to high ambient temperatures for 61 days. As was expected, lambs supplemented with EOD3 alone exhibited greater weight gain, improved feed efficiency, higher carcass weight, and lower intestinal mass than unsupplemented lambs. However, the addition of either *Bacillus subtilis* or virginiamycin to EOD3 did not improve performance beyond that achieved with EOD3 alone.

## 1. Introduction

High ambient temperatures adversely affect livestock productivity [1]. Even in cattle and lambs adapted to hot environments, high ambient temperature (HAT) impairs dry matter intake, nutrient absorption, protein synthesis, and increases maintenance energy requirements, resulting in reduced growth performance and feed efficiency during the fattening phase [2,3]. Therefore, under warm environmental conditions, a range of strategies has been implemented to mitigate the adverse effects of heat stress on the health and productivity of intensive ruminant systems [4,5]. Among these, dietary supplementation with feed additives—used either individually or in combination—has received increasing attention as a promising approach to improve average daily gain and enhance feed and energy efficiency and carcass traits [6]. Notably, a blend of essential oils (EO) and vitamin D_3_ has consistently been shown to improve energy efficiency in ruminants fattening in HAT conditions. Essential oil (EO) compounds, including thymol, limonene, eugenol, and piperine, exhibit ionophore-like and antimicrobial properties that can slow ruminal starch digestion, increase the ruminal propionate-to-acetate molar ratio, and reduce ruminal protein degradation [7,8,9]. In addition, several EO constituents, such as thymol, carvacrol, and limonene, possess potent antioxidant activity by scavenging free radicals, reducing intracellular reactive oxygen species (ROS), and inhibiting lipid peroxidation [10]. Likewise, supplementation with 25-hydroxyvitamin D3 at 0.10–0.12 mg/kg of diet has been associated with improved carcass characteristics, including greater carcass weight and dressing percentage [11,12,13]. Based on these complementary modes of action, supplementation with a blend containing approximately 100 mg/kg of essential oils (thymol, eugenol, vanillin, guaiac, and limonene in different concentrations) and 10 mg/kg of 25-hydroxyvitamin D_3_ significantly increased average daily gain (8.8%), gain-to-feed ratio (5.4%), the observed dietary net energy (3.7%), and carcass weight (4%) in ruminants finished under severe heat stress [temperature–humidity index (THI) > 80] [14,15]. Even so, efforts to maximize the benefits of feed additives continue. Combining feed additives with different modes of action has been shown to enhance their efficacy through synergistic or complementary effects, resulting in greater improvements in animal health and productivity [16]. *Bacillus subtilis* (BS), a probiotic widely used as a feed additive in ruminants and non-ruminant species, modulates the intestinal microbiota by competitively excluding pathogenic microorganisms [17]. In addition to promoting gastrointestinal (GIT) health and modifying ruminal fermentation in a manner analogous to antibiotic feed additives (i.e., monensin; Sarmikasoglou et al. [18]), BS also exerts antioxidant and immunomodulatory effects [19]. These beneficial physiological properties are similar to those observed in animals supplemented with essential oils; thus, it would be expected that BS supplementation could help alleviate the negative impact of HAT on productivity. In non-ruminant species, the use of BS as a feed additive has consistently shown benefits in the productivity of chickens and pigs [20,21]; however, even under favorable environmental conditions, the effect of BS supplementation on growth performance of ruminants has generally been modest [22,23]. While the mechanisms of action of probiotics and essential oils are not fully understood, they appear to act through distinct pathways. Therefore, a combination of probiotic BS with essential oils could have complementary effects. This anticipation was founded on earlier research that shown improved reactions when BS was given in conjunction with monensin, which functions similarly to essential oils [24].

On the other hand, antibiotics that enhance ruminal fermentation, such as virginiamycin, a non-ionophore antibiotic, have been shown to improve growth performance of feedlot cattle under HAT conditions by increasing weight gain and feed efficiency [25,26]. Although virginiamycin and the ionophore antibiotic monensin differ in their mechanisms of action, both selectively inhibit Gram-positive ruminal bacteria [27]. Consequently, their combined supplementation has produced greater improvements in animal performance than either additive alone in both feedlot cattle [28,29,30] and dairy cows [31]. However, the combined use of virginiamycin and monensin is not currently approved for inclusion in animal feeds in many regulatory jurisdictions, limiting its practical application. While combining monensin (MON) with *Bacillus subtilis* (BS) or virginiamycin (VM) has produced positive responses [24,28,30], the same has not been observed when MON is combined with essential oils (EO). Numerous studies have shown that this combination does not improve growth performance or feed efficiency beyond the responses achieved with either additive alone [24,29,32,33]. This lack of additive effects may be explained by the similar mechanisms through which EO and MON modulate ruminal fermentation [7,34]. Therefore, because EO and VM act through distinct mechanisms, combining EOD3 with VM may result in complementary or synergistic effects. Whether this combination can enhance the performance of ruminants fed high-energy diets under high ambient temperature (HAT) conditions remains unknown.

Therefore, we hypothesized that combining BS or VM with EOD3 may increase the magnitude of the productive responses to EOD3 supplementation of lambs receiving a high-energy diet under HAT conditions. To test this hypothesis, a feeding trial was conducted using finishing lambs exposed to high ambient heat load conditions (THI > 80) and fed a high-energy diet (2.08 Mcal NE_m_/kg). Growth performance, dietary energetics, carcass characteristics, shoulder tissue composition, and visceral mass were evaluated.

## 2. Materials and Methods

The study was performed in the Feedlot Lamb Research Unit of Universidad Autónoma de Sinaloa, situated in Culiacán (24°46′13″ N, 107°21′14″ W; 55 m.a.s.l.), Mexico. Culiacán experiences a tropical environment. Throughout the experiment, the mean daily ambient temperature was 30.9 °C, with low and highest temperatures recorded at 27.5 °C and 34.2 °C, respectively, alongside an average relative humidity of 66.7%, with minimum and maximum values of 50.4% and 85.3%, respectively. The Ethics Committee of the School of Veterinary Medicine and Zootechnics of the Autonomous University of Sinaloa approved all animal handling procedures, which adhered to the Federal Guidelines for Animal Use and Care [35] (Protocol #14052025, approved on 2 May 2025).

### 2.1. Climatic Variables and Temperature-Humidity Index (THI) Calculation

Two on-site weather stations (Thermo-hygrometer Avaly, Model DTH880, Mofeg S.A., Zapopan, Jalisco, Mexico) collected hourly data on climate variables, particularly ambient temperature and relative humidity. The formula THI = 0.81 × T + (RH/100) × (T − 14.40) + 46.40 was used to calculate the temperature-humidity index (THI), where RH stands for relative humidity, and T is the temperature in degrees Celsius [36].

### 2.2. Animals, Diet, and Experimental Design

Forty-eight Pelibuey × Katahdin intact lambs (140 ± 9 d of age; initial BW = 33.7 ± 3.2 kg) were used in a randomized complete block design to evaluate the effects of supplementing a blend of essential oils plus vitamin D_3_ (EOD3), either alone or combined with a probiotic or an antibiotic, on growth performance, dietary energy utilization, carcass characteristics, and visceral organ mass of finishing lambs under high ambient temperature (HAT) conditions. Eight weeks before the start of the trial, lambs were transported to the feedlot facilities, clinically examined by a veterinarian, and treated with an antiparasitic agent (Closantil 5%; 5 mg/kg BW; Chinoin Veterinaria, Aguascalientes, Ags., Mexico). During this period, lambs were gradually adapted to the experimental growing-finishing diet. At the beginning of the experiment, lambs were weighed using an electronic scale (TORREY TIL/S:1072691; TORREY Electronics Inc., Houston, TX, USA), blocked by initial BW, and randomly assigned within six weight blocks to one of four dietary treatments (six pens per treatment, two lambs per pen). Pens measured 6 m^2^ and were equipped with overhead shade, a tucuruguay–sand mixed floor, bucket waterers, and 1 m fence-line feed bunks. Lambs were fed the same total mixed growing-finishing diet formulated with a 90:10 concentrate-to-forage ratio, in accordance with the NRC [37] feeding standards (Table 1) for 61 d. The dietary treatments consisted of: (1) a basal diet without feed additives (Control); (2) the basal diet supplemented with EOD3 (100 mg essential oils plus 0.10 mg vitamin D_3_/kg diet); (3) the EOD3 diet plus *Bacillus subtilis* PB6 (2 g/kg diet, providing 2.2 × 10^8^ CFU; EOD3+BS); and (4) the EOD3 diet plus virginiamycin (25 mg/kg diet; EOD3+VM). The EOD3 source used was a product that contains a standardized mixture of essential oils, including (approximate amount of the EO fraction) thymol (25–35%), eugenol (5–10%), vanillin (10–20%), guaiac (10–15%), and limonene (20–35%), plus 25-hydroxyvitamin D3 (Victus Performance; DSM-Firmenich Nutrition and Animal Health, DSM Nutritional Products México, Guadalajara, Jal., Mexico), and to reach the desired concentration, it was included at 200 g/ton of feed. *Bacillus subtilis* PB6 was supplied as CLOSTAT Dry (Kemin Industries, Des Moines, IA, USA), and it was included at 2 kg/ton of feed to reach the target CFU concentration/kg of feed, whereas virginiamycin was supplied as Stafac-500 (Phibro Animal Health Corporation, Guadalajara, Jal., Mexico), and it was included at 50 g/ton of feed to obtain the target concentration of 25 mg VM/kg diet. Dietary treatments were randomly assigned to pens within each weight block. To achieve the desired additive concentrations, each additive was hand-weighed using a precision balance (Ohaus AS612; Pine Brook, NJ, USA), premixed with ground corn and trace mineral salt for 5 min, and then incorporated into the complete diet using a paddle mixer (Model 30910-7, Coyoacán, Mexico) for an additional 7 min. Lambs were offered fresh feed twice daily at 0800 and 1400 h. The morning feeding was restricted to 300 g/lamb, whereas the afternoon feeding was offered ad libitum to maintain feed refusals of approximately 50 g/kg of feed offered. Fresh water was available at all times. Feed refusals were collected daily between 0740 and 0750 h, weighed, and stored for subsequent dry matter (DM) determination. DMI was calculated from the difference between DM offered and DM refused. Dietary samples were collected weekly, composited, and analyzed for DM (method 930.15) and crude protein (method 984.13; N × 6.25) according to AOAC [38], and neutral detergent fiber according to Van Soest et al. [39]. Lambs were weighed before the morning feeding on d 1 and d 61. Initial live weight was multiplied by 0.96 to estimate shrunk body weight (SBW) and minimize variation associated with gastrointestinal fill [40]. Before the final BW measurement, lambs were fasted for 18 h with free access to water.

### 2.3. Calculations of Productive Performance

Average daily gain (ADG) was calculated as the difference between final and initial shrunk body weight (SBW) divided by the number of days on feed. Gain to feed conversion ratio (GF) was calculated as the ratio of ADG to DMI. The efficiency of dietary energy utilization was evaluated by comparing observed dietary net energy (NE), estimated from animal performance, with expected dietary NE based on diet formulation, expressed as the observed-to-expected NE ratio. Expected DMI (kg/d) was calculated as: DMI = (EM/2.08) + (EG/1.41), where 2.08 and 1.41 represent the dietary net energy values for maintenance (NE_m_) and gain (NE_g_), respectively, of the basal diet according to NRC [37]. The energy required for maintenance (EM, Mcal/d) was calculated as EM = 0.056 × SBW^0.75^. Energy required for gain (EG) was estimated according to NRC [41], using the coefficient 0.276, which assumes a mature body weight of 113 kg for intact Pelibuey × Katahdin lambs. The observed dietary NE_m_ concentration was calculated by solving the quadratic equation using the calculated EM and EG requirements together with the observed DMI throughout the experiment.

$$x=\frac{-b\pm \sqrt{b^{2}-4ac}}{2c}$$

where *a* = −0.41EM, *b* = 0.877 EM + 0.41 DMI + EG, *c* = −0.877 DMI, and *x* = observed dietary NE_m_, Mcal/kg [42].

### 2.4. Carcass Characteristics, Shoulder Tissue Composition, and Whole Cuts Data

At the end of the feeding trial, all lambs were slaughtered. After slaughter, the gastrointestinal tract was removed, and the weight of each visceral organ was recorded (g). Carcasses with kidneys and internal fat were chilled at −2 to 1 °C for 24 h. Subsequently, subcutaneous fat thickness, longissimus thoracis (LM) muscle area, and kidney, pelvic, and heart fat (KPH) were measured according to the procedures described by USDA [43] and Quezada-Rubio et al. [44]. The KPH is reported as a percentage of cold carcass weight. To obtain the whole cuts, each carcass was split into two halves. The left side was fabricated into wholesale cuts without trimming, according to the guidelines of the Institutional Meat Purchase Specifications [45]. Neck (IMPS 200), breast (IMPS209), shoulder (IMPS2010), shoulder (IMPS207), rack (IMPS204), and ribs (IMPS209_A) were obtained from the foresaddle, and the loins (IMPS231), leg (IMPS233), and flank (IMPS232) from the hindsaddle. The composition of the shoulder tissues was determined by anatomical dissection following the methodology of Luaces et al. [46].

### 2.5. Visceral Mass Data

The gastrointestinal tract (GIT) was removed from the carcass and weighed full. It was subsequently emptied, washed, and reweighed to determine its digesta-free weight. Empty body weight (EBW) was calculated by subtracting the weight of the gastrointestinal digesta (difference between full and digesta-free GIT weight) from the final shrunk body weight (SBW). All tissue weights are expressed on a fresh-tissue basis. Organ weights are presented as grams of fresh tissue per kilogram of final EBW. Total visceral mass was calculated as the sum of the stomach complex, small intestine, large intestine, liver, lungs, and heart, including gastrointestinal digesta. The weight of the stomach complex was calculated as the sum of the digesta-free weights of the rumen, reticulum, omasum, and abomasum.

### 2.6. Statistical Analysis

All datasets were assessed for normality using the Shapiro–Wilk test on the model residuals. When departures from normality were detected, the residual distributions were further examined to assess the magnitude of the deviation and the potential influence of extreme observations. No substantial departures from normality were identified that warranted data transformation or alternative statistical procedures. Data on growth performance, dietary energetics, carcass characteristics, whole cuts, shoulder muscle tissue, and visceral mass were analyzed as a randomized complete block design with sampling. The analysis used the MIXED procedure of SAS 9.3 software [47]. The pen was the experimental unit, and the animals within it were the sampling unit. The statistical linear model was as follows:

$$Y_{ijk}=\mu +\beta_{i}+\tau_{j}+\varepsilon_{ij}+\delta ijk$$

where *Y_ijk_* is the response variable, *μ* is the general mean or common effect, *β_i_* represents the fixed effect of initial weight as the blocking criterion, *τ_j_* is the fixed effect of treatment, *ε_ij_* is the block-by-treatment interaction, which serves as the random experimental error, and *δ_ijk_* denotes the sampling units within replicates within treatments as random sampling error effects.

The following contrasts were performed: (1) Control vs. EOD3, (2) EOD3 vs. EOD3+BS, and (3) EOD3 vs. EOD3+VM. In addition, treatment means were separated using the “honest significant difference” Tukey’s test (Tukey’s HSD). The least squares means and standard errors are reported in all cases. Contrasts are considered significant when the *p*-value ≤ 0.05.

## 3. Results

It is important to highlight that the present design was specifically intended to determine whether adding BS or VM to the EOD3-containing diet would provide an additional benefit beyond that obtained with EOD3 alone. Therefore, the comparisons of primary interest were EOD3+BS versus EOD3 and EOD3+VM versus EOD3. We must clarify that, because BS-alone and VM-alone treatments were not included, the effects observed with the combination treatments cannot be unequivocally attributed to BS or VM independently, nor can a statistical interaction between EOD3 and either additive be established.

Even though extreme ambient heat conditions were present, no animals were removed from the experiment due to illness or death.

### 3.1. Ambient Temperature

Table 2 summarizes the average ambient temperature and relative humidity (RH) recorded during the experiment. The corresponding average minimum and maximum temperature–humidity index (THI) values were 76.55 and 88.74, respectively, with an overall mean THI of 82.52 ± 1.10, which falls within the Danger category (THI = 79–84) [48]. During the total 61 experimental days, the daily maximum THI exceeded 80 for an average of 5:20 h/day, indicating prolonged exposure to hot environmental conditions that could adversely affect feed intake, energy metabolism, and growth performance [49].

### 3.2. Growth Performance and Dietary Energy

The growth performance and dietary net energy data are shown in Table 3. Compared to non-supplemented lambs, lambs that received EOD3 alone showed greater (*p* < 0.03) average daily gain (ADG; 15.3%), with a similar DMI (*p* = 0.46); therefore, the gain-to-feed ratio (GF) and the observed-to-expected dietary NE ratio were increased by 12.3% and 5.9%, respectively. The combination of EOD3 with BS resulted in identical ADG (0.230 kg) but a numerically larger DMI (7.1%; *p* = 0.087) than the EOD3 treatment, leading to lower GF (*p* = 0.024) and a lower observed-to-expected dietary NE ratio (*p* < 0.01). Similarly, combining VM with EOD3 did not increase growth response (*p* = 0.69) or modified DMI (*p* = 0.29); thus, GF (*p* = 0.49) and dietary energy utilization efficiency were numerically slightly lower (2.2%, *p* = 0.09) to the lambs that were fed with EOD3 alone.

### 3.3. Carcass Traits, Shoulder Tissue Composition, Whole Cuts and Visceral Mass

Treatment effects on carcass traits, shoulder tissue composition, and whole cuts are presented in Table 4. Lambs that were supplemented with EOD3 alone or in combination with BS and VM showed heavier carcass weight (4.4%, *p* < 0.04) than the unsupplemented lambs; this difference was because supplemented lambs showed heavier final weights than lambs that did not receive supplemental additives. Supplementing additives alone or combined did not modify the dressing percentage, LM area, KPH, or fat thickness. Similarly, shoulder tissue composition and whole cuts (expressed as percentage of CCW) were not modified by additive supplementation.

Lambs that received EOD3 and its combinations had 8.0% lower relative intestinal mass (g intestines/kg EBW) than controls (*p* < 0.04), without affecting the other visceral mass components evaluated, including visceral fat depots (Table 5).

## 4. Discussion

Exposure to high ambient temperature (HAT) during long-term periods does not necessarily induce physiological alterations indicative of heat stress. Long-term studies have indicated that cattle maintained under HAT conditions (THI > 79) exhibit adaptive changes in feed intake and energy utilization without significant alterations in physiological stress indicators, such as rectal temperature, respiratory rate, or blood metabolite concentrations [50]. Although this adaptive response, referred to as “adaptive physiology,” enables animals to cope with prolonged heat exposure, it incurs an energetic cost that can impair growth performance and reduce the efficiency of dietary energy utilization [2,51], even among animals adapted to hot climates, including hairy-breed lambs and their crosses [52] and Zebu cattle and their crosses [53]. Therefore, the objective of the present study was to determine whether combining selected feed additives with EOD3 supplementation could enhance the productive responses of lambs fed a high-energy diet under HAT conditions.

### 4.1. Effects of Supplemental EOD3 on Performance and Carcass Traits

Based on the average DMI and BW recorded during the experiment, the estimated daily EOD3 intake was equivalent to 3.03 mg/kg BW. This dosage falls within the range (3–5 mg/kg BW) previously reported to improve growth performance, dietary energy utilization, and carcass characteristics in ruminants fed high-energy diets under high ambient temperature (HAT) conditions [14,15]. Information regarding the effect of EOD3 on DMI is limited; however, EOD3 generally does not modify DM intake compared with control or antibiotic-supplemented groups under either HAT [15,54] or favorable ambient conditions [55].

Consistent with the present findings, Mendoza-Cortez et al. [14] reported that HAT-adapted crossbred cattle (50% Zebu) fed a moderate-energy diet (1.99 Mcal NEm/kg) under an average THI of 82.7 and supplemented with EOD3 (3 mg/kg BW) had 8.5% greater ADG than cattle receiving 24 mg monensin sodium/kg diet, a widely used ionophore antibiotic for improving feed efficiency and mitigating the adverse effects of HAT [56]. The greater ADG was associated with a 5% numerical increase in DMI, whereas feed efficiency and dietary energy utilization improved by 4.0% and 2.5%, respectively. Similarly, Estrada-Angulo et al. [15] reported that Pelibuey × Katahdin lambs fed a high-energy diet (2.10 Mcal NEm/kg) under HAT conditions (average THI = 80.4) and supplemented with EOD3 at 5 mg/kg BW had 9.2% greater ADG and 6.7% greater feed efficiency than unsupplemented controls, without affecting DMI.

Because daily net energy intake was similar between control and EOD3 lambs in the present experiment (2.25 vs. 2.32 Mcal NEm/day), the greater ADG observed with EOD3 likely resulted from more efficient utilization of dietary energy rather than greater energy intake. Essential oils such as thymol, eugenol, vanillin, guaiac, and limonene have been associated with changes in ruminal fermentation, nutrient utilization, and oxidative status [7,8,9,10], processes that can be affected by HAT [57,58,59]. Likewise, vitamin D3 supplementation at rates similar to those used in the present study has been associated with greater protein retention and lean tissue deposition [12,60], including under subtropical conditions [61]. Thus, these effects may have contributed to the improved ADG and feed efficiency observed with EOD3. However, because ruminal fermentation, oxidative status, nutrient metabolism, and protein deposition were not measured, these mechanisms remain speculative and their contribution to the present response cannot be established.

The observed-to-expected dietary net energy (NE) ratio provides an indicator of the efficiency with which dietary energy was utilized relative to the value predicted from diet formulation and observed animal performance. A ratio of 1.0 indicates agreement between observed and expected dietary NE [40], whereas values below or above 1.0 indicate lower or greater energy utilization than expected, respectively. In the present study, control lambs showed 10.1% lower efficiency (0.899), whereas EOD3 lambs showed a 4.8% reduction (0.952) relative to expected dietary energy. A reduction in energy efficiency in non-supplemented lambs under HAT conditions is consistent with previous reports [62,63] and may reflect the additional energetic cost associated with physiological adaptation to heat load [64]. Therefore, the smaller reduction observed in EOD3 lambs may indicate greater efficiency of dietary energy utilization under HAT conditions.

Previous studies have shown that essential oils alone generally have little or no effect on whole cuts, tissue composition, or carcass characteristics, including dressing percentage, longissimus muscle (LM) area, and subcutaneous fat thickness, in feedlot cattle [8,65,66] or finishing lambs [67,68,69]. Likewise, the effects of vitamin D3 on carcass development remain inconsistent. A dietary inclusion of 0.10 mg vitamin D3/kg diet provides approximately 4000 IU/kg diet, equivalent to twice the vitamin D3 allowance recommended by the NRC [37]. Nelson et al. [70] suggested that vitamin D3 supplementation above conventional recommendations may improve livestock health and productivity. However, evidence for long-term effects of supranutritional vitamin D3 supplementation on carcass characteristics remains variable. Carvalho and Perdigão [12] reported that 0.10 mg vitamin D3/kg diet for 90 days increased carcass weight but did not affect dressing percentage or LM area, whereas Acedo et al. [13] observed improved dressing percentage without changes in carcass weight or LM area in Nellore cattle receiving the same supplementation rate for at least 90 days. Reported improvements in carcass traits have been attributed to enhanced protein accretion associated with elevated vitamin D3 supplementation [60]. Information regarding the influence of the essential oil–vitamin D3 blend (EOD3) on carcass characteristics is also limited and remains inconclusive. Among the few published studies, two reported no significant effects of EOD3 on dressing percentage, LM area, or fat thickness, tissue composition, and whole cuts, which is consistent with the findings of the present study [15,54]. In contrast, Estrada-Angulo et al. [55] observed an increase in LM area following EOD3 supplementation, although dressing percentage, whole cuts, and tissue composition were not altered. Whereas twofold OED3 dosage supplementation (200 mg/kg diet) in Holstein steers finished during 285 d with a high-energy diet (2.20 Mcal EN_m_/kg diet) reduced carcass fat thickness without effects on other carcass characteristics [33].

Previous studies in both ruminants [67,68] and non-ruminant species [71,72] have consistently reported that supplementation with essential oils (EO) alone decreases the relative intestinal mass (g/kg EBW). A comparable response has also been observed when EO is administered in combination with vitamin D3 [15,54], suggesting that the inclusion of D3 does not alter this effect. Although the underlying biological mechanism remains uncertain, it has been proposed that EO may reduce intestinal mass through antibiotic-like actions that decrease epithelial thickness [73].

### 4.2. Effects of Combining Supplemental EOD3 with BS

The use of direct-fed microbials (DFMs) has gained considerable attention as a strategy to enhance productivity and gastrointestinal health in ruminants while reducing reliance on in-feed antibiotics. Although their application in feedlot production is well established and has been practiced for several decades [74], the mechanisms responsible for their beneficial effects are not yet fully understood. Proposed modes of action include modulation of the microbial communities inhabiting the rumen and hindgut, improvements in nutrient digestion and utilization, and regulation of immune function at the gastrointestinal mucosal interface [74,75].

Among the commercially available DFMs, CLOSTAT^®^ (Kemin Industries, Des Moines, IA, USA) contains *Bacillus subtilis* PB6 (BS), a spore-forming bacterium that remains dormant in the rumen but germinates in the small and large intestines in response to acidic conditions and bile salts [76]. Supplementation with BS has been shown to reduce Salmonella populations in the gastrointestinal tract and feces of ruminants [77], enhance intestinal barrier integrity and gastrointestinal health [78], and exert both antioxidant and immunomodulatory effects [19]. Despite these physiological benefits, improvements in growth performance of feedlot ruminants have generally been limited, even under optimal production conditions [22,23,79]. Considering the complementary biological activities of BS and the essential oil blend included in EOD3, we hypothesized that their combined supplementation would produce additive or synergistic effects. This expectation was based on previous findings showing enhanced responses when BS was administered together with monensin [24], which acts similarly to essential oils with a slight different modes of action [80]. Information on the combined use of probiotics and essential oils (EO) in ruminants remains limited. Nevertheless, the available evidence suggests that certain probiotic–EO combinations may produce complementary or synergistic effects. For example, supplementation of post-weaning calves with a probiotic mixture containing *Lactobacillus acidophilus*, *Enterococcus faecium*, and *Saccharomyces cerevisiae*, together with an EO blend derived from walnuts, olives, soybeans, lavender, eucalyptus, coconut, orange, peppermint, and sesame seeds, enhanced both average daily gain and feed efficiency [81].

More evidence for beneficial probiotic–EO interactions has been reported in non-ruminant species, particularly with *Bacillus subtilis*. In breeder broilers, the combination of *B. subtilis* (1 × 10^9^ CFU) and an EO blend containing cinnamaldehyde, thymol, and piperine improved reproductive performance [82]. Likewise, dietary supplementation of weaned pigs exposed to high ambient temperatures with 1 g of *B. subtilis* plus 300 mg/kg of a thymol–carvacrol EO blend increased average daily gain [83]. However, in the present experiment, supplementing *Bacillus subtilis* in combination with EOD3 decreased dietary energy efficiency by 5.4% relative to EOD3 alone. Evidence regarding the combined use of *B. subtilis* and essential oils (EO) in finishing ruminants is limited. Estrada et al. [24] evaluated dietary supplementation with *B. subtilis* PB6 (2.2 × 10^8^ CFU/kg diet), an EO blend containing thymol, carvacrol, and cinnamaldehyde (300 mg/kg diet), and monensin (28 mg/kg diet) in finishing lambs raised under favorable environmental conditions. None of the additive combinations improved growth performance compared with the control treatment. The authors concluded that the lack of a productive response was more likely associated with interactions between the EO blend and monensin than with incompatibility between EO and *B. subtilis*. In contrast, Podversich et al. [84] reported that supplementing Charolais × Angus steers fed a corn silage-based diet with a mixture of *Bacillus licheniformis* and *Bacillus subtilis* plus monoterpene- and phenylpropene-based essential oils (200 mg/kg diet) for 83 days reduced the observed-to-expected dietary net energy ratio by 4.2% compared with steers supplemented with monensin alone. This reduction closely resembles the 5.4% decrease observed in the present study when comparing EOD3 + *B. subtilis* with EOD3 alone. Although the available evidence remains limited, the consistency between these findings suggests that the response to combinations of probiotics and essential oils may depend on the bacterial strain used or the type of essential oil with which it was combined. The mechanisms underlying this apparent lack of additivity remain unclear, highlighting the need for additional studies to elucidate interactions among feed additives and to identify supplementation strategies that are effective across different production and environmental conditions.

The lack of treatment effects on carcass characteristics and wholesale cuts is consistent with previous reports indicating that probiotic supplementation, like essential oil supplementation, exerts little influence on these traits in feedlot lambs [67,85,86,87,88,89,90]. Similarly, while both additives have been reported to reduce intestinal wall thickness by attenuating inflammatory responses and promoting the expression of protective proteins [91], combining them did not result in an additive or synergistic response [54].

### 4.3. Effects of Combining Supplemental EOD3 with VM

Virginiamycin exerts its antimicrobial activity by inhibiting bacterial protein synthesis through the synergistic action of two components: Factor M, a peptolide, and Factor S, a macrocyclic lactone. This dual mechanism primarily targets Gram-positive bacteria [92], resulting in improved ruminal fermentation efficiency and productivity efficiency when is supplemented at rate of 25–28 mg/kg diet [26,93]. In contrast, essential oils (EO), similarly to the ionophore monensin, mainly affect bacterial membrane integrity by increasing membrane permeability and promoting the loss of potassium ions and other intracellular constituents, with their antimicrobial activity being directed predominantly against Gram-positive microorganisms [94]. These alterations in the ruminal microbial ecosystem are associated with more efficient fermentation and improved energy utilization. Both monensin and virginiamycin have consistently been shown to enhance dietary energy utilization and productive performance in feedlot cattle, including animals exposed to high ambient heat load [56,93]. Because these additives differ in their modes of antimicrobial action, their simultaneous supplementation frequently produces complementary responses, leading to greater improvements in growth performance and dietary energy efficiency than either additive alone [28,29,30,95]. Nevertheless, the combined inclusion of monensin and virginiamycin is prohibited in animal feeds in many regulatory jurisdictions, restricting its practical application. Considering that EO share several antimicrobial characteristics with monensin, it was hypothesized that replacing the ionophore with EO while maintaining virginiamycin supplementation could provide comparable complementary effects without regulatory limitations. Under this premise, combining EO with VM was expected to further improve dietary energy utilization in lambs finished under HAT conditions. Contrary to this hypothesis, however, supplementation with EOD3 plus VM failed to improve growth performance, carcass characteristics, or dietary energy utilization beyond the responses obtained with EOD3 alone. The interaction between EO and antibiotics is known to be highly dependent on the specific compounds involved. Several studies have demonstrated synergistic antimicrobial effects when bioactive EO constituents such as carvacrol, cinnamaldehyde, and eugenol are combined with conventional antibiotics, including ampicillin, ciprofloxacin, and erythromycin [96]. Conversely, these same EO compounds, either individually or as blends, have also exhibited antagonistic interactions with other antibiotics, including gentamicin and tobramycin [97]. Information regarding potential interactions between EO and streptogramin antibiotics, such as virginiamycin or quinupristin/dalfopristin, remains extremely limited. Therefore, although the EOD3-VM combination failed to generate additive productive responses in the present study, it likewise showed no evidence of antagonism. This contrasts with previous reports in which EO combined with monensin negatively affected productive responses in finishing cattle [33,79] and lambs [24].

The observed-to-expected dietary net energy ratio further supports these findings. Relative to formulated dietary energy values, unsupplemented lambs exhibited an 10.1% reduction in observed dietary NE, whereas lambs receiving EOD3 alone showed a smaller reduction of only 4.8%, while supplementation with EOD3 in combination with VM resulted in slightly higher reductions of 6.8%. Consequently, under the conditions of this experiment, VM did not potentiate the beneficial effect of EOD3 on dietary energy utilization during exposure to elevated THI. Because this represents the first study evaluating the combination of EO and virginiamycin in finishing lambs subjected to HAT conditions, additional research is required to determine whether these findings are consistent across different dietary formulations, EO compositions, antimicrobial inclusion rates, and environmental conditions.

The current experiment showed that supplementation with EOD3 alone had no effect on dressing percentage, longissimus muscle area, subcutaneous fat thickness, shoulder tissue composition, or wholesale cut yields; thus, the absence of effects of EOD3 and VM on carcass traits is not surprising. The above is because previous studies have shown that virginiamycin supplementation alone rarely alters carcass characteristics [26,55,98].

EOD3+VM treatment reduced intestinal visceral mass to an extent comparable with EOD3 alone, while no additional effects were detected on the remaining visceral organ measurements.

## 5. Conclusions

The primary objective of this study was to determine whether supplementation with the probiotic *Bacillus subtilis* (BS) or the non-ionophore antibiotic virginiamycin (VM) could enhance the beneficial effects of a blend of essential oils combined with vitamin D_3_ (EOD3), a nutritional strategy intended to mitigate the adverse effects of high ambient heat load on the productive performance of finishing lambs. Relative to the formulated dietary energy values, unsupplemented lambs exhibited an 10.1% reduction in observed dietary net energy (NE) utilization, a decrease that was nearly identical to that observed in lambs receiving EOD3 plus BS (10.0%). In contrast, lambs supplemented with EOD3 alone showed a markedly smaller reduction (4.8%), whereas those receiving EOD3 plus VM exhibited an intermediate reduction (6.8%). Likewise, growth performance, carcass characteristics, and dietary energy utilization were either similar to or slightly lower in lambs receiving EOD3 combined VM than in those supplemented with EOD3 alone. Combining EOD3 with BS increased DMI but negatively affected the efficacy of EOD3 to improve dietary energy utilization. These findings indicate that, under the conditions of the present study, neither BS nor VM enhanced the beneficial effects of EOD3 on dietary energy utilization during exposure to elevated thermal load. In contrast, supplementation with EOD3 alone consistently improved average daily gain, feed efficiency, dietary NE utilization, and carcass weight compared with the unsupplemented control, confirming its effectiveness as a nutritional strategy for finishing lambs exposed to heat stress. Because this is the first study to evaluate the combined use of essential oils and virginiamycin in finishing lambs under high ambient heat load, further research involving larger populations is warranted to determine whether these responses are consistent across different dietary formulations, essential oil compositions, antimicrobial inclusion rates, and environmental conditions.

## Figures and Tables

**Table 1:** Composition of dietary treatments offered to lambs.

Item	Additives			
	None	EOD3	EOD3+BS	EOD3+VM
Ingredient composition (g/kg DM basis)				
Dry-rolled corn	670	670	670	670
Sudan grass hay	100	100	100	100
Soybean meal	127	127	127	127
Tallow	20	20	20	20
Blend of essential oil plus vitamin D3 ^1^	----	+++	+++	+++
*Bacillus subtilis* ^2^	----	----	+++	----
Virginiamycin ^3^	----	----	----	+++
Molasses cane	50	50	50	50
Mineral-vitamin premix	10	10	10	10
Urea	8	8	8	8
Limestone	15	15	15	15
Dry Matter (%)	88.73	88.73	88.73	88.73
Chemical composition, (DM basis) ^4^				
Total crude protein (%)	15.50	15.50	15.50	15.50
Ether extract (%)	5.08	5.08	5.08	5.08
NDF (%)	15.73	15.73	15.73	15.73
ADF (%)	6.87	6.87	6.87	6.87
Calculated net energy (Mcal/kg) ^5^				
Maintenance	2.08	2.08	2.08	2.08
Gain	1.41	1.41	1.41	1.41

The symbol “----” means no inclusion of additives; the symbol “+++” means inclusion of the additive. ^1^ Supplement included at a rate of 200 mg/kg of diet which provides a mixture of essential oils 100 mg/kg diet DM plus 0.10 mg/kg diet DM of 25-hydroxy-vitamin-D_3_ (Victus Performance_;_ DSM Nutritional Products, Basel, Switzerland). ^2^ Supplement included at a rate of 2 g/kg diet of a product containing *Bacillus subtilis* 2.2 × 10^8^ CFU (CLOSTAT dry, Kemin Industries, Des Moines, IA, USA). ^3^ Supplement included at a rate of 50 mg of a product/kg diet which provides 25 mg virginiamycin/kg diet (Stafac-500; Phibro Animal Health Corporation, Guadalajara, Jalisco, México). ^4^ Mineral-vitamin supplement contained (for each 10 kg of supplement): CoSO_4_, 2 g; CuSO_4_, 41 g; FeSO_4_, 143 g; ZnO, 32 g; MNSO_4_, 44 g; KI, 2 g; NaCl, 3750 g; vitamin A, 400,000 UI; vitamin D, 18.75 UI; CaCO_3_, 5948 g. ^5^ Based on tabular values for individual feed ingredients [37] with the exception of DM, CP and NDF, which were determined in our laboratory.

**Table 2:** Ambient temperature (Ta), relative humidity (RH) and calculated temperature-humidity index (THI) ^1^ registered every hour and expressed as a weekly average.

Week	Mean T_a_ (°C)	Max T_a_ (°C)	Min T_a_ (°C)	Mean RH (%)	Max RH (%)	Min RH (%)	Mean THI ^1^	Max THI	Min THI
1	29.23 ± 1.3	30.94 ± 1.7	27.53 ± 1.1	69.25 ± 5.5	78.26 ± 3.8	60.24 ± 8.2	80.37 ± 1.3	84.44 ± 2.3	76.55 ± 1.4
2	30.92 ± 0.9	32.99 ± 1.0	28.85 ± 1.2	66.13 ± 5.2	75.29 ± 5.4	56.98 ± 5.3	82.40 ± 1.1	87.15 ± 0.9	77.78 ± 1.5
3	31.69 ± 1.1	33.62 ± 1.2	29.75 ± 0.9	60.55 ± 6.8	69.45 ± 7.7	51.64 ± 6.2	85.54 ± 0.9	86.99 ± 3.3	78.12 ± 0.8
4	31.93 ± 0.9	34.07 ± 0.4	29.79 ± 1.0	60.42 ± 5.5	70.36 ± 5.7	50.48 ± 4.8	82.88 ± 0.7	87.87 ± 0.5	78.02 ± 1.1
5	32.17 ± 0.5	34.20 ± 3.4	30.14 ± 0.8	62.50 ± 3.4	77.33 ± 4.0	51.67 ± 3.2	83.63 ± 1.1	88.69 ± 1.0	78.63 ± 1.2
6	31.48 ± 0.9	33.77 ± 1.0	29.18 ± 0.9	64.60 ± 3.3	77.17 ± 3.4	52.02 ± 3.2	82.97 ± 1.0	88.74 ± 1.2	77.49 ± 1.5
7	30.94 ± 1.2	32.88 ± 1.4	28.99 ± 1.3	70.04 ± 2.6	79.57 ± 1.9	60.50 ± 3.6	83.10 ± 1.8	87.81 ± 1.7	78.52 ± 1.9
8	30.52 ± 0.8	32.54 ± 0.9	28.51 ± 1.1	70.98 ± 4.4	81.19 ± 5.0	60.76 ± 4.8	82.62 ± 1.0	87.54 ± 1.2	77.88 ± 1.5
9 *	29.75 ± 0.9	31.36 ± 0.9	28.14 ± 1.1	75.72 ± 2.2	85.30 ± 4.1	66.136 ± 5.0	82.18 ± 1.2	86.35 ± 1.5	78.17 ± 1.8
Mean	30.96 ± 1.0	32.93 ± 1.6	28.99 ± 1.1	66.69 ± 4.2	76.66 ± 4.6	56.71 ± 4.5	82.52 ± 1.1	87.29 ± 1.3	77.91 ± 1.4

^1^ THI = 0.81 × ambient temperature + [(relative humidity × (ambient temperature − 14.4)] + 46.4. THI code (normal THI < 74; alert 75 to 79; danger 79 to 84; and emergency > 84) [48]. * This corresponds to the records from 5 days of the last week of the experiment.

**Table 3:** Effect of different dietary additives supplemented alone or combined on growth performance and dietary energy in finishing lambs.

Item	Additives ^1^				SEM		*p*-Value	
	None	EOD3	EOD3+BS	EOD3+VM		Control vs. EOD3	EOD3 vs. EOD_3_+BS	EOD3 vs. EOD_3_+VM
Days on test	61	61	61	61				
Pen replicates	6	6	6	6				
Live weight (kg) ^2^								
Initial	33.59	33.69	33.74	33.82	0.489	0.88	0.99	0.99
Final	44.34 ^b^	46.09 ^a^	46.09 ^a^	46.47 ^a^	0.567	0.04	0.99	0.64
Average daily gain (kg)	0.176 ^b^	0.203 ^a^	0.203 ^a^	0.207 ^a^	0.007	0.02	0.94	0.70
Dry matter intake (kg/d)	1.084 ^b^	1.118 ^ab^	1.198 ^a^	1.165 ^ab^	0.031	0.43	0.09	0.29
Gain to feed (kg/kg)	0.162 ^c^	0.182 ^a^	0.170 ^bc^	0.178 ^ab^	0.003	<0.01	0.02	0.46
Observed dietary NE (Mcal/kg)								
Maintenance	1.870 ^b^	1.981 ^a^	1.872 ^b^	1.940 ^a^	0.014	<0.01	<0.01	<0.07
Gain	1.230 ^b^	1.327 ^a^	1.231 ^b^	1.292 ^a^	0.012	<0.01	<0.01	<0.07
Observed to expected dietary Net energy ratio								
Maintenance	0.899 ^b^	0.952 ^a^	0.900 ^b^	0.932 ^a^	0.007	<0.01	<0.01	<0.07
Gain	0.872 ^b^	0.941 ^a^	0.873 ^b^	0.916 ^a^	0.009	<0.01	<0.01	<0.07

^a,b,c^ Means in the same row with different superscript letters differ *p* ≤ 0.05. ^1^ EOD3 = A mixture of essential oils 100 mg/kg diet DM plus 0.10 mg/kg diet DM of 25-hydroxy-vitamin-D_3_ (Victus Performance_;_ DSM Nutritional Products, Basel, Switzerland); BS = *Bacillus subtilis* 2.2 × 10^8^ CFU/g (CLOSTAT dry, Kemin Industries, Des Moines, IA, USA); VM = 25 mg virginiamycin/kg diet (Stafac-500; Phibro Animal Health Corporation, Guadalajara, Jalisco, México). ^2^ Initial live weight (LW) was reduced by 4% to adjust for the gastrointestinal fill. Final LW was obtained after an 18 h fast without access to feed (drinking water was not restricted).

**Table 4:** Effect of different dietary additives supplemented alone or combined on carcass characteristics, tissue composition, and whole cuts in long-term finishing lambs.

Item	Additives ^1^				SEM		*p*-Value	
	None	EOD3	EOD3+BS	EOD3+VM		Control vs. EOD3	EOD3 vs. EOD_3_+BS	EOD3 vs. EOD_3_+VM
Hot carcass weight (kg)	25.32 ^b^	26.44 ^a^	26.37 ^a^	26.48 ^a^	0.341	0.04	0.88	0.93
Dressing percentage	57.08	57.37	57.07	56.93	0.534	0.71	0.70	0.57
Cold carcass weight (kg)	24.95 ^b^	26.06 ^a^	25.94 ^a^	26.10 ^a^	0.336	0.04	0.88	0.93
LM area (cm^2^)	14.28	14.87	14.34	14.27	0.500	0.41	0.46	0.41
Fat thickness (cm)	0.190	0.161	0.180	0.182	0.016	0.20	0.38	0.35
Kidney-pelvic fat	4.33	4.63	4.21	4.53	0.186	0.27	0.13	0.69
Shoulder composition (%)								
Muscle	67.35	67.05	67.31	67.20	0.390	0.59	0.64	0.81
Fat	14.72	14.66	14.21	14.49	0.296	0.60	0.50	0.99
Bone	17.93	18.29	18.48	18.31	0.352	0.49	0.70	0.96
Muscle: fat ratio	4.57	4.57	4.73	4.64	0.115	0.87	0.51	0.94
Muscle: bone ratio	3.75	3.66	3.64	3.67	0.086	0.50	0.84	0.99
Whole cuts (as percentage of CCW)								
Forequarter IMPS202	41.59	41.12	41.16	40.81	0.415	0.42	0.94	0.59
Hindquarter IMPS230	36.30	35.76	36.46	36.17	0.359	0.30	0.19	0.44
Neck	9.45	10.09	9.99	9.71	0.272	0.12	0.82	0.35
Shoulder IMPS206	8.89	8.57	8.41	8.41	0.258	0.40	0.67	0.67
Shoulder IMPS207	14.57	14.41	14.48	14.26	0.192	0.58	0.81	0.56
Rack IMPS204	6.82	6.92	6.87	6.70	0.103	0.46	0.69	0.13
Breast IMPS209	4.50	4.58	4.63	4.55	0.179	0.78	0.85	0.92
Ribs IMPS 209A	6.81	6.92	6.87	6.69	0.146	0.46	0.69	0.13
Loin IMPS231	6.98	6.97	7.03	7.05	0.117	0.96	0.72	0.61
Flank IMPS232	6.39	6.22	6.32	6.52	0.159	0.45	0.66	0.21
Leg IMPS233	22.90	22.67	23.09	22.50	0.229	0.35	0.09	0.68

^a,b^ Means in the same row with different superscript letters differ *p* ≤ 0.05. ^1^ EOD3 = A mixture of essential oils 100 mg/kg diet DM plus 0.10 mg/kg diet DM of 25-hydroxy-vitamin-D_3_ (Victus Performance; DSM Nutritional Products, Basel, Switzerland); BS = *Bacillus subtilis* 2.2 × 10^8^ CFU/g (CLOSTAT dry, Kemin Industries, Des Moines, IA, USA); VM = 25 mg virginiamycin/kg diet (Stafac-500; Phibro Animal Health Corporation, Guadalajara, Jalisco, México).

**Table 5:** Effect of different dietary additives supplemented alone or combined on relative visceral organ mass.

Item	Additives ^1^				SEM		*p*-Value	
	None	EOD3	EOD3+BS	EOD3+VM		Control vs. EOD3	EOD3 vs. EOD_3_+BS	EOD3 vs. EOD_3_+VM
GIT fill (kg)	4.24	4.27	4.75	4.66	0.201	0.93	0.11	0.07
Empty body weight (% of full weight)	90.38	90.70	89.58	89.66	0.646	0.63	0.11	0.09
Organs (g/kg, empty body weight)								
Stomach complex	24.43	23.06	23.97	24.24	0.556	0.22	0.42	0.28
Intestines	41.41 ^a^	37.90 ^b^	38.21 ^b^	38.12 ^b^	0.547	0.03	0.45	0.86
Liver	16.12	15.33	15.38	16.31	0.423	0.20	0.94	0.13
Heart/lungs	21.04	20.86	21.16	20.85	0.417	0.87	0.77	0.98
Kidney	2.47	2.60	2.63	2.72	0.059	0.11	0.72	0.16
Omental fat	31.84	32.67	33.30	32.80	1.882	0.76	0.81	0.96
Mesenteric fat	15.41	14.73	13.24	14.64	0.941	0.62	0.28	0.95
Visceral fat	47.26	47.40	46.54	47.44	2.381	0.97	0.80	0.99

^a,b^ Means in the same row with different superscript letters differ *p* ≤ 0.05. GIT = gastrointestinal tract. ^1^ EOD3 = A mixture of essential oils 100 mg/kg diet DM plus 0.10 mg/kg diet DM of 25-hydroxy-vitamin-D_3_ (Victus Performance_;_ DSM Nutritional Products, Basel, Switzerland); BS = *Bacillus subtilis* 2.2 × 10^8^ CFU/g (CLOSTAT dry, Kemin Industries, Des Moines, IA, USA); VM = 25 mg virginiamycin/kg diet (Stafac-500; Phibro Animal Health Corporation, Guadalajara, Jalisco, México).

## Data Availability

The data that support the findings of this study are available from the corresponding authors upon reasonable request.

## References

[1] Habebb A.A., Gad A.E., Atta M.A. (2018). Temperature-humidity index as indicators to stress of climatic conditions with relation to production and reproduction of farm animals. Int. J. Biotechnol. Res. Adv..

[2] Lees A.M., Sejian V., Wallage A.L., Steel C.C., Mader T.L., Lees J.C., Gaughan J.B. (2019). The Impact of Heat Load on Cattle. Animals.

[3] McManus C.M., Lucci C.M., Maranhão A.Q., Pimentel D., Pimentel F., Rezende P.S. (2022). Response to Heat Stress for Small Ruminants: Physiological and Genetic Aspects. Livest. Sci..

[4] Dean L., Tarpoff A.J., Nickles K., Place S., Edwards-Callaway L. (2023). Heat Stress Mitigation Strategies in Feedyards: Use, Perceptions, and Experiences of Industry Stakeholders. Animals.

[5] Kotsampasi B., Karatzia M.A., Tsiokos D., Chadio S. (2024). Nutritional Strategies to Alleviate Stress and Improve Welfare in Dairy Ruminants. Animals.

[6] Jongbo A.O., de Borba L.P., Pereira R.M.M., Bello Q.O., Gregoratto L.L., de Souza D.P., Adeyeye O.A., Vieira F.M.C. (2026). Heat stress in livestock under tropical climates: Impacts and mitigation strategies. Trop. Anim. Health Prod..

[7] Samii S.S., Wallace N., Nagaraja T.G., Engstrom M.A., Miesner M.D., Armendariz C.K., Titgemeyer E.C. (2016). Effects of limonene on ruminal Fusobacterium necrophorum concentrations, fermentation, and lysine degradation in cattle. J. Anim. Sci..

[8] Meschiatti M.A.P., Pellarin L.A., Batalha C.D.A., Acedo T.S., Tamassia L.F.M., Cortinhas C.S., Gouvea V.N.D., Santos F.A.P., Dórea J.R. (2016). Effects of essential oils and exogenous enzymes on intake, digestibility, and rumen fermentation in finishing Nellore cattle. J. Anim. Sci..

[9] Nasir M., Rodríguez-Prado M., Simoni M., Martín-Orúe S.M., Pérez J.F., Calsamiglia S. (2025). Optimizing Essential Oil Mixtures: Synergistic Effects on Cattle Rumen Fermentation and Methane Emission. Animals.

[10] Huang Y., Ebrahimi H., Barselli E., Foti M.C., Amorati R. (2026). Essential Oils as Antioxidants: Mechanistic Insights from Radical Scavenging to Redox Signaling. Antioxidants.

[11] Gouvêa V.N., Meschiatti M.A.P., Moraes J.M.M., Batalha C.D.A., Dórea J.R.R., Acedo T.S., Tamassia L.F.M., Owens F.N., Santos F.A.P. (2019). Effects of alternative feed additives and flint maize grain particle size on growth performance, carcass traits and nutrient digestibility of finishing beef cattle. J. Agri. Sci..

[12] Carvalho V.V., Perdigão A. (2019). Supplementation of 25-hydroxy-vitamin-D3 and increased vitamin E as a strategy to increase carcass weight of feedlot beef cattle. J. Anim. Sci..

[13] Acedo T.S., de Gouvêa V.N., Vasconcellos G.M., Arrigoni M., Martins C.L., Millen D.D., Muller L.R., Melo G.F., Rizzieri R.A., Costa C.F. (2018). Effect of 25-hydroxy-vitamin-D3 on feedlot cattle. J. Anim. Sci..

[14] Mendoza-Cortéz D.A., Ramos-Méndez J.L., Arteaga-Wences Y., Félix-Bernal A., Estrada-Angulo A., Castro-Pérez B.I., Urías-Estrada J.D., Barreras A., Zinn R., Plascencia A. (2022). Influence of a supplemental blend of essential oils plus 25-hydroxyvitamin-d3 on feedlot cattle performance during the early-growing phase under conditions of high ambient temperature. Indian J. Anim. Res..

[15] Estrada-Angulo A., Verdugo-Insúa M., Escobedo-Gallegos L.d.G., Castro-Pérez B.I., Urías-Estrada J.D., Ponce-Barraza E., Mendoza-Cortez D., Ríos-Rincón F.G., Monge-Navarro F., Barreras A. (2024). Influences of a supplemental blend of essential oils plus 25-hydroxy-vit-d3 and zilpaterol hydrochloride (β2 agonist) on growth performance and carcass measures of feedlot lambs finished under conditions of high ambient temperature. Animals.

[16] Radzikowski D. (2017). Effect of probiotics, prebiotics and synbiotics on the productivity and health of dairy cows and calves. World Sci. News..

[17] Zhang X., Cao J., Han S.Z., Liu Z.L., Cui J.Y., Zhang Y.H., Xiong H.S., Wang X.L. (2025). *Bacillus subtilis*: Applications in the livestock and poultry industry in recent years: Review. Anim. Biosci..

[18] Sarmikasoglou E., Sumadong P., Dagaew G., Johnson M.L., Vinyard J.R., Salas-Solis G., Siregar M., Faciola A.P. (2024). Effects of *Bacillus subtilis* on in vitro ruminal fermentation and methane production. Transl. Anim. Sci..

[19] Jung H.-S., Lee H.-W., Kim K.-T., Lee N.-K., Paik H.-D. (2024). Anti-inflammatory, antioxidant effects, and antimicrobial effect of *Bacillus subtilis* P223. Food Sci. Biotechnol..

[20] Zhang S.Z., Zhong G.S., Shao D.W., Wang Q., Hu Y., Wu T., Ji C., Shi S. (2021). Dietary supplementation with *Bacillus subtilis* promotes growth performance of broilers by altering the dominant microbial community. Poult. Sci..

[21] Li R., Liu J., Liu Y., Cao L., Qiu W., Quin M. (2023). Probiotic Effects of *Bacillus subtilis* on Growth Performance and Intestinal Microecological Balance of Growing-to-Finishing Pigs. J. Food Biochem..

[22] Dias B.G.C., Santos F.A.P., Meschiatti M., Brixner B.M., Almeida A.A., Queiroz O., Cappellozza B.I. (2022). Effects of feeding different probiotic types on metabolic, performance, and carcass responses of *Bos indicus* feedlot cattle offered a high-concentrate diet. J. Anim. Sci..

[23] Smith Z.K., Broadway P.R., Underwood K.R., Rushe W.C., Walker J.A., Sanchez N.C.B., Carroll J.A., Lafleur D., Hergenreder J.E. (2021). Evaluation of *Bacillus subtilis* PB6 on feedlot phase growth performance, efficiency of dietary net energy utilization, and fecal and subiliac lymph node Salmonella prevalence in spring placement yearling beef steers fed in southeastern South Dakota. Transl. Anim. Sci..

[24] Estrada-Angulo A., Escobedo-Gallegos L.d.G., Arteaga-Wences Y.J., Ramos-Méndez J.L., Quezada-Rubio J.A., Vizcarra-Chávez C.A., Valdés-García Y.S., Barreras A., Zinn R.A., Plascencia A. (2023). Effect of Combining the Ionophore Monensin with Natural Antimicrobials Supplemented in the Last Phase of Finishing of Lambs: Growth Performance, Dietary Energetics, and Carcass Characteristics. Animals.

[25] Montano M., Manriquez O.M., Salinas-Chavira J., Torrentera N., Zinn R.A. (2015). Effects of monensin and virginiamycin supplementation in finishing diets with distiller dried grains plus solubles on growth performance and digestive function of steers. J. Anim. Appl. Res..

[26] Salinas-Chavira J., Barreras A., Plascencia A., Montano M.F., Navarrete J.D., Torrentera N., Zinn R.A. (2016). Influence of protein nutrition and virginiamycin supplementation on feedlot growth-performance and digestive function of calf-fed Holstein steer. J. Anim. Sci..

[27] Nagaraja T.G., Taylor M.B. (1987). Susceptibility and resistance of ruminal bacteria to antimicrobial feed additives. Appl. Environ. Microbiol..

[28] da Fonseca M.P., Borges A.L.d.C.C., Carvalho P.H.d.A., de Silva R.R., Goncalves L.C., Borges I., Lage H.F., Ferreira A.L., Saliba E.O.S., Jayme D.G. (2019). Energy partitioning in cattle fed diets based on tropical forage with the inclusion of antibiotic additives. PLoS ONE.

[29] Heker J.C., Neumann M., Ueno R.K., Falbo M.K., Galbeiro S., de Souza A.M., Venancio B.J., Santos L.C., Askel E.J. (2018). Effect of monensin sodium associative to virginiamycin and/or essential oils on the performance of feedlot finished steers. Semin. Ciênc. Agrár. Londrina.

[30] Ceconi I., Viano S.A., Méndez D.G., González L., Davies P., Elizalde J.C., Bressan E., Grandini D., Nagaraja T.G., Tedeschi L.O. (2022). Combined use of monensin and virginiamycin to improve rumen and liver health and performance of feedlot-finished steers. Transl. Anim. Sci..

[31] Erasmus L.J., Muya C., Erasmus S., Coertze R.F., Catton D.G. (2008). Effect of virginiamycin and monensin supplementation on performance of multiparous Holstein cows. Livest. Sci..

[32] Araujo R.C., Daley D.R., Goodall S.R., Jalil S., Guimares-Bisnieto O.A., Bude A.M., Wagner J., Engle T. (2019). Effects of a microencapsulated blend of essential oils supplemented alone or in combination with monensin on performance and carcass characteristics of growing-finishing beef steers. Appl. Anim. Sci..

[33] Latack B.C., Carvalho P.H.V., Zinn R.A. (2022). The interaction of feeding an eubiotic blend of essential oils plus 25-hydroxy-vit-D3 on performance, carcass characteristics, and dietary energetics of calf-fed Holstein steers. Front. Vet. Sci..

[34] Koyunco M., Canbolat O. (2010). Effect of carvacrol on intake, rumen fermentation, growth performance and carcass characteristics of growing lambs. J. Appl. Anim. Res..

[35] (1995). Normas Oficiales Mexicanas. Diario Oficial de la Federación. (NOM-051-ZOO-1995, NOM-033-ZOO-1995) Trato Humanitario de Animales de Producción, de Compañía y Animales Silvestres Durante el Proceso de Crianza, Desarrollo de Experimentos, Movilización y Sacrificio. https://dof.gob.mx/nota_detalle.php?codigo=4870842&fecha=23/03/1998#gsc.tab=0.

[36] Dikmen S., Hansen P.J. (2009). Is the temperature-humidity index the best indicator of heat stress in lactating dairy cows in a subtropical environment?. J. Dairy Sci..

[37] National Research Council (2007). Nutrient Requirements of Small Ruminants: Sheep, Goats, Cervids, and New World Camelids.

[38] Association of Official Analytical Chemists (2000). Official Method of Analysis.

[39] Van Soest P.J., Robertson J.B., Lewis B.A. (1991). Methods for Dietary Fiber, Neutral Detergent Fiber, and Nonstarch Polysaccharides in Relation to Animal Nutrition. J. Dairy Sci..

[40] Cannas A., Tedeschi L.O., Fox D.G., Pell A.N., Van Soest P.J. (2004). A mechanistic model for predicting the nutrient requirements and feed biological values for sheep. J. Anim. Sci..

[41] National Research Council (1985). Nutrient Requirements of Sheep.

[42] Zinn R.A., Barreras A., Owens F.N., Plascencia A. (2008). Performance by feedlot steers and heifers: ADG, mature weight, DMI and dietary energetics. J. Anim. Sci..

[43] Agricultural Marketing Service (USDA) (1992). Official United States Standards for Grades of Carcass Lambs, Yearling Mutton and Mutton Carcasses.

[44] Quezada-Rubio J.A., Estrada-Angulo E.A., Castro-Pérez B.I., Urías-Estrada J.D., Ponce-Barraza E., Escobedo-Gallegos L.d.G., Mendoza-Cortez D., Barreras A., Carrillo-Muro O., Plascencia A. (2026). Effect of Combining a Prebiotic (Autolyzed Yeast from Saccharomyces cerevisiae) and Probiotic (*Bacillus subtilis*) Added in a High-Energy Diet on Growth Performance, Dietary Energetics, and Carcass Traits of Fattening Hairy Lambs. Animals.

[45] Institutional Meat Purchase Specifications (IMPS) (2014). For Fresh Lamb and Mutton Serie 200.

[46] Luaces M.L., Calvo C., Fernández B., Fernández A., Viana J.L., Sánchez L. (2019). Predicting equation for tisular composition in carcass of Gallega breed lambs. Arch. Zoot..

[47] Statistical Analytical System, Institute Inc. (2004). SAS Proprietary Software Release 9.3.

[48] Mader T.L., Davis M.S., Brown-Brandl T. (2006). Environmental factors influencing heat stress in feedlot cattle. J. Anim. Sci..

[49] Silanikove N. (2000). Effects of heat stress on the welfare of extensively managed domestic ruminants: A review. Livest. Prod. Sci..

[50] Renaudeau D., Collin A., Yahav S., de Basilio V., Gourdine J.L., Collier R.J. (2012). Adaptation to hot climate and strategies to alleviate heat stress in livestock production. Animals.

[51] Moreno J.A., Lopes de Sá A., Cohelo C.F., Pereira R.N., Batista E.D., Ladeira M.M., Casagrande D.R., Gionbelli M.P. (2021). Effect of heat stress on ingestive, digestive, ruminal and physiological parameters of Nellore cattle feeding low- or high-energy diets. Livest. Sci..

[52] Rosas-Rodríguez M., Serna-Lagunes R., Salinas-Ruiz J., Ayala-Rodríguez J.M., Cárdenas B.A.P., Salazar-Ortiz J. (2022). Growth performance and carcass classification of pure Pelibuey and crossbred lambs raised under an intensive production system in a warm-humid climate. Rev. Mex. Cienc. Pecu..

[53] Hansen P.J. (2004). Physiological and cellular adaptations of zebu cattle to thermal stress. Anim. Reprod. Sci..

[54] Escobedo-Gallegos L.d.G., Estrada-Angulo A., Castro-Pérez B.I., Urías-Estrada J.D., Calderón-Garay E., Ramírez-Santiago L., Valdés-García Y.S., Barreras A., Zinn R.A., Plascencia A. (2023). Essential Oils Combined with Vitamin D3 or with Probiotic as an Alternative to the Ionophore Monensin Supplemented in High-Energy Diets for Lambs Long-Term Finished under Subtropical Climate. Animals.

[55] Estrada-Angulo A., Mendoza-Cortéz D.A., Ramos-Méndez J.L., Arteaga-Wences Y., Urías-Estrada J.D., Castro-Pérez B.I., Ríos-Rincón F.G., Rodríguez-Gaxiola M.A., Barreras A., Zinn R.A. (2022). Comparing Blend of Essential Oils Plus 25-Hydroxy-Vit-D3 Versus Monensin Plus Virginiamycin Combination in Finishing Feedlot Cattle: Growth Performance, Dietary Energetics, and Carcass Traits. Animals.

[56] Barreras A., Castro-Pérez B.I., López-Soto M.A., Torrentera N.G., Montaño M.F., Estrada-Angulo A., Ríos F.G., Dávila-Ramos H., Plascencia A., Zinn R.A. (2013). Influence of ionophore supplementation on growth performance, dietary energetics and carcass characteristics in finishing cattle during period of heat stress. Asian-Australas. J. Anim. Sci..

[57] Bedford A., Beckett L., Harthan L., Wang C., Jiang N., Schramm H., Guan L.L., Daniels K.M., Hanigan M.D., White R.R. (2020). Ruminal volatile fatty acid absorption is affected by elevated ambient temperature. Sci. Rep..

[58] Guo Z., Gao S., Ding J., He J., Ma L., Bu D. (2022). Effects of Heat Stress on the Ruminal Epithelial Barrier of Dairy Cows Revealed by Micromorphological Observation and Transcriptomic Analysis. Front. Genet..

[59] Zhang Q., Yang L., Li Y., Gu P., Si R., Zhu L., Zhang W. (2025). Heat stress affects dairy cow performance via oxidative stress, hypothalamic–pituitary–adrenal axis, gut microbiota, and multi-dimensional mitigation. Front. Vet. Sci..

[60] Martins T.E., Acedo T.S., Gouvêa V.N., Vasconcellos G.M., Arrigoni M., Martins C.L., Millen D.D., Pai M.D., Perdigão A., Melo G.F. (2020). Effects of 25-hydroxycholecalciferol supplementation on gene expression of feedlot cattle. J. Anim. Sci..

[61] Martins T.E., Gouvêa V.N., Perdigão A., Niehues M.B., Martins C.L., Millen D.D., Acedo T.S., Carvalho V.V., Tamassia L.F.M., Arrigoni M.D.B. (2025). Effects of supplemental 25-hydroxyvitamin D3 on growth performance, physiological responses, and gene expression of skeletal muscle growth of finishing beef cattle. J. Anim. Sci..

[62] National Research Council (1981). Effect of Environment on Nutrient Requirements of Domestic Animals (NRC).

[63] Vicente-Pérez V.R., Macías-Cruz U., Avendaño-Reyes L., Correa-Calderón A., López-Vaca M.A., Lara-Rivera A.L. (2020). Heat stress impacts in hair sheep production. Rev. Mex. Cienc. Pecu..

[64] Macías-Cruz U., Valadez-García K.M., López-Baca M.d.l.Á., Avendaño-Reyes L., Vicente-Pérez R., Mellado M., Meza-Herrera C.A., Roque-Jiménez J.A., Díaz-Molina R., Luna-Nevárez P. (2025). Environmental, Physiological, Metabolic, and Growth Factors Defining the Presence of Oxidative Stress in Feedlot Hair Lambs Subjected to Heat Stress. Ruminants.

[65] Wilson H.C., Hilscher F.H., Boyd B.M., Watson A.K., McDonald J.C., Erickson G.E. (2020). Impact of essential oils blend on beef cattle performance and carcass characteristics in diets with increasing corn silage inclusions. Neb. Beef Cattle Rep..

[66] Souza M.M., Vargas J.A.C., Carvalho A.M., Conti A.C.M., Tavares D.H.S., Coelho B.P.L., Santos E.P., Neiva J.N.M., Miotto F.R.C. (2026). The Impact of Oregano Essential Oil and the Finishing System on Performance, Carcass Characteristics and Meat Quality in Heifers. Ruminants.

[67] Arteaga-Wences Y., Estrada-Angulo A., Gerardo Ríos-Rincón F.G., Castro-Pérez B.I., Mendoza-Cortéz D.A., Manriquez-Núñez O.M., Barreras A., Corona-Gochi L., Zinn R.A., Perea-Domínguez X.P. (2021). The effects of feeding a standardized mixture of essential oils vs. monensin on growth performance, dietary energy, and carcass characteristics of lambs fed a high-energy finishing diet. Small Rum. Res..

[68] Estrada-Angulo A., Arteaga-Wences Y.J., Castro-Pérez B.I., Urías-Estrada J.D., Gaxiola-Camacho S., Angulo-Montoya C., Ponce-Barraza E., Barreras A., Corona L., Zinn R.A. (2021). Blend of Essential Oils Supplemented Alone or Combined with Exogenous Amylase Compared with Virginiamycin Supplementation on Finishing Lambs. Animals.

[69] Cavalheiro E.R., Caetano W.F., da Silva K.D., Rodrigues E.R., Ferreira G.A., Parra A.R.P., Mizubuti I.Y., Simonelli S.M., Do Prado R.M., Calixto O.P.P. (2026). Effects of garlic and rosemary essential oils on growth performance, carcass traits, and meat quality of lambs fed high-concentrate diets. Small Rum. Res..

[70] Nelson C.D., Reinhardt T.A., Lippolis J.D., Sacco R.E., Nonnecke B.J. (2012). Vitamin D signaling in the bovine immune system: A model for understanding human vitamin D requirements. Nutrients.

[71] Wang H., Liang S., Li X., Xiaojun Yang X., Long F., Xin Yang X. (2019). Effects of encapsulated essential oils and organic acids on laying performance, egg quality, intestinal morphology, barrier function, and microflora count of hens during the early laying period. Poult. Sci..

[72] Ge C., Luo X., Lv Y., Wu L., Hu Z., Huang W., Zhan S., Shen X., Hui C., Yu D. (2024). Essential oils ameliorate the intestinal damages induced by nonylphenol exposure by modulating tryptophan metabolism and activating aryl hydrocarbon receptor via gut microbiota regulation. Chemosphere.

[73] Ghazanfari S., Mohammadi Z., Adib-Moradi M. (2015). Effects of coriander essential oil on the performance, blood characteristics, intestinal microbiota and histological of broilers. Braz. J. Poult. Sci..

[74] McAllister T.A., Beauchemin K.A., Alazzeh A.Y., Baah J., Teather R.M., Stanford K. (2011). Review: The use of direct fed microbials to mitigate pathogens and enhance production in cattle. Can. J. Anim. Sci..

[75] Krehbiel C.R., Rust S.R., Zhang G., Gilliland S.E. (2003). Bacterial direct-fed microbials in ruminant diets: Performance response and mode of action. J. Anim. Sci..

[76] Lin A.S.H., Teo A.Y.-L., Meng T.H. (2007). Antimicrobial Compounds from *Bacillus subtilis* for Use Against Animal and Human Pathogens. U.S. Patent.

[77] Broadway P.R., Carroll J.A., Burdick Sanchez N.C., Callaway T.R., Lawhon S.D., Gart E.V., Bryan L.K., Nisbet D.J., Hughes H.D., Legako J.F. (2020). *Bacillus subtilis* PB6 supplementation in weaned Holstein steers during an experimental Salmonella challenge. Foodborne Pathog. Dis..

[78] Sun W., Chen W., Meng K., Cai L., Li G., Li X., Jiang X. (2023). Dietary Supplementation with Probiotic *Bacillus licheniformis* S6 Improves Intestinal Integrity via Modulating Intestinal Barrier Function and Microbial Diversity in Weaned Piglets. Biology.

[79] Eyre K.E., Pan L., Harper K., Silva L.F.P. (2025). The effect of a Bacillus-based probiotic on feed intake and digestibility of a forage and a feedlot diet in Bos indicus steers. Vet. Anim. Sci..

[80] Lima A.B., de Almeida K.L., Lima B.E.T.d., Haddi K., Passetti L.C.G., Rosado G.L., Bento C.B.P. (2025). Evaluation of Plant Essential Oils as Natural Alternatives to Monensin in *In Vitro* Ruminal Fermentation. Fermentation.

[81] Gading B.M.W.T., Agus A., Irawan A., Panjono P. (2020). Growth performance, hematological and mineral profile of post—Weaning calves as influenced by inclusion of pelleted-concentrate supplement containing essential oils and probiotics. Iran. J. Appl. Anim. Sci..

[82] Liu X., Chen Y.B., Tang S.G., Deng Y.Y., Xiao B., He C.Q., Guo S.C., Zhou X., Qu X. (2021). Dietary encapsulated *Bacillus subtilis* and essential oil supplementation improves reproductive performance and hormone concentrations of broiler breeders during the late laying period. Livest. Sci..

[83] Tan B.F., Lim T., Boontiam W. (2021). Effect of dietary supplementation with essential oils and a Bacillus probiotic on growth performance, diarrhoea and blood metabolites in weaned pigs. Anim. Prod. Sci..

[84] Podversich F., Ribeiro T.L.M., Francis F.L., Rusche W.C., Smith Z.K.F. (2025). Evaluation of dietary inclusion of a blend of essential oils and probiotics for growing beef cattle. Transl. Anim. Sci..

[85] Dorantes-Iturbide G., Orzuna-Orzuna J.F., Lara-Bueno A., Mendoza-Martínez G.D., Miranda-Romero L.A., Lee-Rangel H.A. (2022). Essential Oils as a Dietary Additive for Small Ruminants: A Meta-Analysis on Performance, Rumen Parameters, Serum Metabolites, and Product Quality. Vet. Sci..

[86] Verdugo-Insúa M., Castro-Pérez I., Urías-Estrada D., Ponce-Barraza E., Ríos-Rincón F., Escobedo-Gallegos L., Estrada-Angulo A., Carrillo-Muro O., Barreras A., Plascencia A. (2026). Dietary supplementation with a zilpaterol and natural additive blend on cut yield and meat quality of fattening hair lambs under conditions of high ambient temperature. Austral J. Vet. Sci..

[87] Whitley N.C., Cazac D., Rude B.J., Jackson-O’Brien D., Parveen S. (2009). Use of a commercial probiotic supplement in meat goats. J. Anim. Sci..

[88] Zerby H.N., Bard J.L., Loerch S.C., Kuber P.S., Radunz A.E., Fluharty F.L. (2011). Effects of diet and Aspergillus oryzae extract or Saccharomyces cerevisiae on growth and carcass characteristics of lambs and steers fed to meet requirements of natural markets. J. Anim. Sci..

[89] Khalid M.F., Shahzad M.A., Sarwar M., Rehman A.U., Sharif M., Mukhtar N. (2011). Probiotics and lamb performance: A review. Afr. J. Agr. Res..

[90] Estrada-Angulo A., Zapata-Ramírez O., Castro-Pérez B.I., Urías-Estrada J.D., Gaxiola-Camacho S., Angulo-Montoya C., Ríos-Rincón F., Barreras A., Zinn R.A., Leyva-Morales J.B. (2021). The effects of single or combined supplementation of probiotics and prebiotics on growth performance, dietary energetics, carcass traits, and visceral mass in lambs finished under subtropical climate conditions. Biol. J..

[91] García-Díaz T., Branco A.F., Jacovaci F.A., Jobim C.C., Daniel J.L.P., Bueno A.V., Ribeiro M.G. (2018). Use of live yeast and mannanoligosacharides in grain-based diets for cattle: Ruminal parameters, nutrient digestibility, and inflammatory response. PLoS ONE.

[92] Chinali G., Moreau P., Cocito C.G. (1981). The Mechanism of Action of Virginiamycin M on the Binding of Aminoacyl-tRNA to Ribosomes Directed by Elongation Factor Tu. Eur. J. Biochem..

[93] Navarrete J.D., Montano M.F., Raymundo C., Salinas-Chavira J., Torrentera N., Zinn R.A. (2017). Effect of energy density and virginiamycin supplementation in diets on growth performance and digestive function of finishing steers. Asian Australas. J. Anim. Sci..

[94] Chouhan S., Sharma K., Guleria S. (2017). Antimicrobial Activity of Some Essential Oils—Present Status and Future Perspectives. Medicines.

[95] Nuñez A.J.C., Caetano M., Berndt A., Demarchi J.J.A.A., Leme P.R., Lanna D.P.D. (2013). Combined use of ionophore and virginiamycin for finishing Nellore steers fed high concentrate diets. Sci. Agric..

[96] Caballero-Gómez N., Manetsberger J., Lavilla-Lerma L., Martínez-Martos J.M., Benomar N., Abriouel H. (2024). Exploring synergistic effects of essential oils compounds with antibiotics and biocides against multidrug-resistant foodborne pathogens. Appl. Food Res..

[97] Moussaoui F., Alaoui T. (2016). Evaluation of antibacterial activity and synergistic effect between antibiotic and the essential oils of some medicinal plants. Asian Pac. J. Trop. Biomed..

[98] Mejía-Delgadillo M.A., Leyva-Medina K.H., Sánchez-Pérez J.N., Lee-Rangel H.A., López-Inzunza H.J., Martínez-León R., Gámez G.M., Covarrubias J.L.P., Ramos H.D., Estrada J.C.R. (2025). Effects of zilpaterol hydrochloride and virginiamycin on growth performance, carcass characteristics, and visceral organ mass in feedlot lambs. Anim. Biosci..

